# Estimation of Recycled Concrete Aggregate’s Water Permeability Coefficient as Earth Construction Material with the Application of an Analytical Method

**DOI:** 10.3390/ma12182920

**Published:** 2019-09-10

**Authors:** Wojciech Sas, Justyna Dzięcioł, Andrzej Głuchowski

**Affiliations:** Water Centre Laboratory, Faculty of Civil and Environmental Engineering, Warsaw University of Life Sciences, 02-787 Warsaw, Poland (W.S.) (A.G.)

**Keywords:** statistical analysis, estimation, permeability, constant head method, estimation coefficient of permeability, recycled concrete aggregate

## Abstract

Creating models based on empirical data and their statistical measurements have been used for a long time in the economic sciences. Increasingly, these methods are used in the technical sciences, such as construction and geotechnical engineering. This allows for reducing the costs of geotechnical research at the design stage. This article presents the research carried out on Recycled Concrete Aggregate (RCA) material with is reclaimed crushed concrete rubble. Permeability tests were carried out using the constant head method. Tests were conducted on blends of RCA with the following particle size ranges: 0.02–16 mm, 0.05–16 mm, 0.1–16 mm, and 0.2–16 mm. The gradients used during the tests were between 0.2 to 0.83, which corresponds to gradients encountered in earth construction and are below the critical gradient. Directly from the tests, the flux velocity for the range of tested gradients were calculated based on filtered water volume measurements. The values of the permeability coefficient (*k*) were then recalculated. Finally, statistical methods were used to determine which physical parameters of the tested material affect the permeability coefficient. The physical parameters selected from the statistical analysis were used to create a model describing the phenomenon. The model can be used to determine the permeability coefficient for a mixed RCA material. The article ends with conclusions and proposals concerning the use of models and the limits of their applicability.

## 1. Introduction

The construction industry was one of the most strongly affected industries during the 2008 global financial crisis in Europe. However, local construction markets have been steadily strengthening since then, which is visible mainly in the countries of Western and Northern Europe. It is forecasted that the total growth of Northwestern and Southern Europe markets will average 2.5% per annum for 2018–2022 [1]. For the construction industry in Central and Eastern Europe, which after the economic crisis is trying to catch up with the rest of Europe, the expected higher market growth is estimated at an average of 4.4% for 2018–2022 [1,2]. 

Despite these optimistic forecasts, the industry faces numerous problems. The most important are the falling profitability of investments and the increase in employee remuneration costs. This is the reason why it is so important to look for solutions that help reduce the cost of construction projects. One of the options is to reduce material costs by using recycled materials [2,3].

Aggregates are materials commonly used in civil engineering. Natural aggregates are mainly used as materials in earth constructions, such as dams, embankments, or other earth structures, e.g., road bases; therefore, the demand for this material is very high [4]. Natural aggregates represent approximately 88% of the demand in the market [4]. Problems with the sustainable development of the natural aggregates market, and with waste management as well, forces engineers to use anthropogenic aggregates in earth structures. The mechanical and physical characteristics of recycled aggregates vary from natural aggregates and need to be further investigated. Better knowledge of their response to different kind of loadings or the permeability characteristics will allow for wider application of this kind of material by designers and engineers [5].

## 2. Literature Review

The proper assessment of soil properties and their parameters is very important in geotechnical modeling. A well-chosen model allows one to save time and reduce the costs of construction investment [6]. However, for the model to provide reliable results, several conditions must be met. The first is to have a large database to assess and recognize the case [7]. Another important requirement is to determine the appropriate physical parameters that should be included in the model. It is also important to determine the nature of the function distribution for empirical research. The last stage, after creating the model, is to determine the parameters of its application. This is due to every model being created on the basis of empirical research that has the character of partial research [7,8].

The central pillar of statistical inference is the analysis of source data from empirical research, based on theorems of probability theory. Properly carried out inference leads to the creation of a model that maps the distribution of empirical data [9,10]. Generalization of the results of statistical observation to the whole phenomenon is carried out using statistical estimation or verification of the hypothesis. The model is created after estimation of the study population parameters’ distributions, based on the observed results. By analyzing the interrelationships between different material properties, the hypotheses and the correctness of the initial assumptions are verified [10]. 

Proper planning of laboratory tests is the key to obtaining a reliable model. The selection of the research sample, followed by statistical analysis, should be based on the knowledge of the phenomenon and the previous research reports [11,12].

The calculation of the probability assumes randomness of the analyzed sample. Often, especially in the scientific research process, randomness is disturbed by study restrictions or is even impossible to achieve due to the limited ability to test the material properties. This is due to measurement errors during the test, e.g., destruction of the test material. The above-mentioned limitations are strictly connected with statistical sample construction and can be considered directed or expert. It leads to the need for representative sample estimation for a given study or phenomenon [13].

The credibility of the model is important from the points of view of the entrepreneur and the researcher. It limits cost and time expenditure. It is also important to evaluate the parameters for the possible substitution of laboratory tests on the analytical method of estimation. This article presents a way to replace laboratory tests of the permeability coefficient by estimating with the use of statistical tools [14,15,16]. 

Reclaimed concrete is widely used in earthworks, especially in road substructures. This allows for its recycling and the reduction of the number of landfills and concrete debris, which can be reused after mechanical crushing. It also reduces the need for natural aggregates in earth construction. Crushed concrete is classified as a material suitable for auxiliary foundations, basic foundations, and cut-off layers [17,18]. A special form of this kind of material application is used as a filtration layer for the construction of levees and dams.

Recycled concrete aggregates (RCAs) are obtained as a result of the crushing process, excluding brick and soft materials, which are then used to obtain residual concrete with a grain size of 0 to 63 mm [19]. RCAs can be used as aggregate for earth construction. They are mainly used in road engineering, in which their geotechnical parameters (strength and deformation susceptibility) have already been recognized [19,20].

The determination of the angle of internal friction (*φ*) for RCAs with gradation sandy gravel (saGr) has already been studied by Sas et al. [21] and Soból et al. [19], who confirmed the results of the test presented by O’Mahony [22]. This is important that RCAs are characterized by a complicated structure and can give the effect of cohesion in non-adhesive soil, which improves the mechanical properties of RCAs [23]. When utilizing RCAs in road engineering constructions and in designing dams and shafts, the permeability coefficient is of particular importance. In the case of natural aggregates, in order to reduce investment costs via replacement of laboratory methods, predictions regarding the hydraulic conductivity based on porosity or grain size distribution are calculated [24]. This is possible because natural aggregates are characterized by the presence of rounded grains, composed mainly of quartz origin with a low roughness. RCA aggregates are rougher and have an irregular shape, which has a significant impact on the water flow and the surface area [25]. 

The significant difference when comparing RCAs with natural aggregates is the remains of hydrated cement on the surface of aggregate grains. As a result, this property leads to a lower specific density of the grains, differentiation in aggregate quality, and a higher absorption ability [26,27].

The porosity of both types of materials are different. In the case of natural aggregates, it is about 3%, and for RCA, it is about 15% [28]. On the surface of RCA aggregates, there is a residue of cement mortar, which affects the ability of the aggregate to absorb water [29,30]. During one day of carrying, the water absorption by RCA increased by 2.56% [30], which is important for the permeability parameters of this material.

Recycled aggregates used in earth constructions are usually cheaper than the natural aggregates. Re-use of construction waste is an environmentally friendly solution. The growing popularity of this material contributes to a better understanding of the properties of this material. Understanding its limitations and recommendations regarding its use already at the stage of the design, then improves the construction works [31].

## 3. Materials and Methods 

### 3.1. Material

The material used for the tests came from the demolition site of a building and was crushed using an impact crusher. The class of concrete strength was evaluated based on laboratory tests. The results of the tests showed that the tested RCA was concrete with a strength of class from C16/20 to C30/35.

The aggregates were 99% composed from broken cement concrete, the rest being of the bulk mass, e.g., glass and brick (Σ(*Rb*, *Rg*, *X*) ≤ 1% m/m), in accordance with the standard EN 933-11:2009 [32,33,34], and contained no asphalt or tar elements. 

Recycling concrete aggregate is regarded as an environmental safety material to use in road pavement and road construction. Maia et al. [35] studied many articles about the chemical analysis of RCA from the last few years. After a review on leaching tests, the obtained information has shown that critical compounds of RCAs are chromium, sulfate, antimony, and selenium. The concentration of these elements should be periodically monitored but the threat of their elevated concentration is very low due to the fact that these elements exist naturally in soil. Leaching of these elements from RCA and concentration in the soil are rare and usually does not exceed existing standards of acceptability. Rodrigues P. et al. [36] more in our research take were notice of supervening and concentration heavy metals and anion in RCA. She suggested that materials should be tested for heavy metals and anions before re-use. Using the laboratory leaching method, one can estimate the actual amount of elements that are eluted into the soil. This allows one to evaluate the ecotoxicity of the material before using it [36,37,38,39]. The norm for other elements, such as cadmium (Cd), mercury (Hg), and lead (Pb), are not exceeded in RCA material [36,40]. 

The pH value is of great importance for the intensity of element leaching. The higher the acidity, the greater the leaching [41]. This is important information from the point of view of practical application.

The material was fractioned to the appropriate fractions according to Galvín et al. [39], and then the RCAs were divided into four blends: 0.02–16 mm, 0.05–16 mm, 0.1–16 mm, and 0.2–16 mm. Each blend was composed of obtained fractions. The grain gradation curve was adopted with respect to the Polish technical standard and was placed between the upper and lower grain grading limits. The resulting mixtures are suitable for earth structures such as dams and embankments [33].

**Figure 1:**
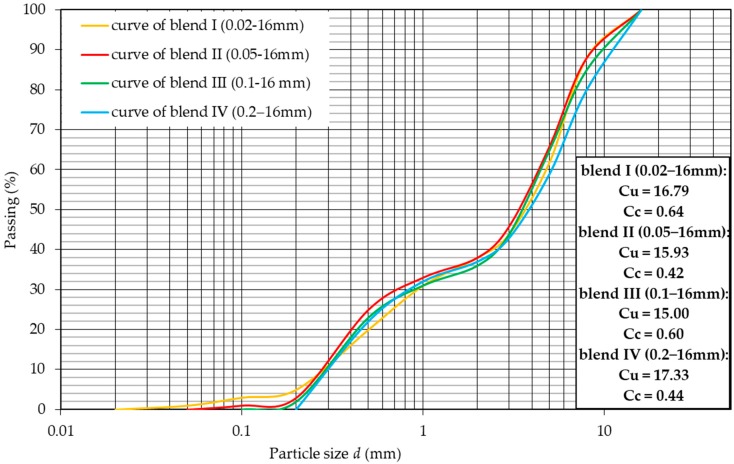
Grain size distribution curve of each tested RCA blends.

**Figure 2:**
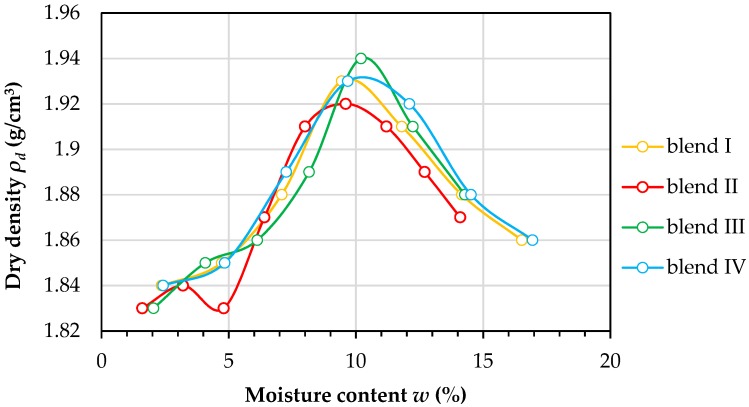
Results of the Proctor tests for RCA blends with an energy density of compaction Ec equal to 0.59 J/cm^3^.

A series of tests were carried out on the obtained blends to determine their physical properties. According to Eurocode, the tested material was classified on the basis of sieve analysis as sandy gravel (saGr) [34]. Figure 1 presents the graining curves for the tested blends, where the particle size distribution from 0 mm to 16 mm is in the standard range for aggregates used as an auxiliary base and improved substrate in road engineering and in earth structures [33]. The coefficient of curvature (C_C_) and coefficient of uniformity (C_U_) were calculated in order to classify the shape of the grading curve of tested blends. The C_U_ value was in the range from 15.0 to 17.33 and C_C_ was in the range from 0.42 to 0.64. The soil was therefore classified as medium-graded according to the Eurocode [34]. For the four blends, the Proctor tests were performed in order to establish the optimum moisture content of the RCAs. The tests were conducted with the energy density of the compaction equal to 0.59 J/cm^3^ and the results are presented in Figure 2.

Results of the Proctor tests indicated that the RCA blends had an optimum moisture contents between 9.4% and 10.2% (9.44%, 9.6%, 10.2%, and 9.6%, respectively, for blends I, II, III, and IV). The maximum dry density ρ_d_ max was between 1.92 and 1.94 g/cm^3^. The RCAs were compacted in the optimum moisture content in a permeability mold to achieve the maximum dry density. These kinds of conditions represent the state in which the subbase soils would be in. The quality of soil samples in permeability tests was measured with the use of the compaction index *IS*, which is the quotient of the soil dry density in the permeability mold *ρ_d,m_* to the maximum dry density for tested blend *ρ_d,max_* (*IS* = *ρ_d,m_/ρ_d,max_*). The RCAs sample quality was qualified based on this quotient. In this study, the samples had an IS parameter in the range of 0.98 to 1.01. Samples below an *IS* equal to 0.98 were excluded from this study.

### 3.2. Permeability Test

**Figure 3:**
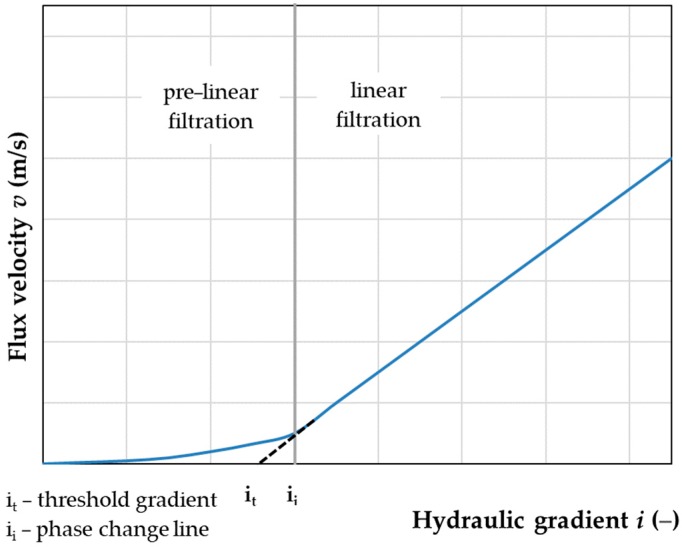
Pre-linear and linear filtration process.

In poor and very poor permeability soils, filtration can only be initiated after a certain hydraulic gradient (*i_0_*) has occurred. This means that the graph *v* = f(*i*) does not come out at the beginning of the hydraulic gradient axis, and even in the initial period, shows curvature caused by a gradual increase in permeability. This filtration is called pre-linear filtration (Figure 3). In order to initiate the movement of the pore water, the threshold stress (τ_0_) must be overcome by the tangential stress (τ) [42,43,44].

In this study, the constant head method test was used to estimate the RCAs’ permeability characteristics. The method is characterized by simplicity and unchanging test conditions, and the constant head method alone is one of the most reliable techniques for measuring permeability in non-cohesive soil [45].

**Figure 4:**
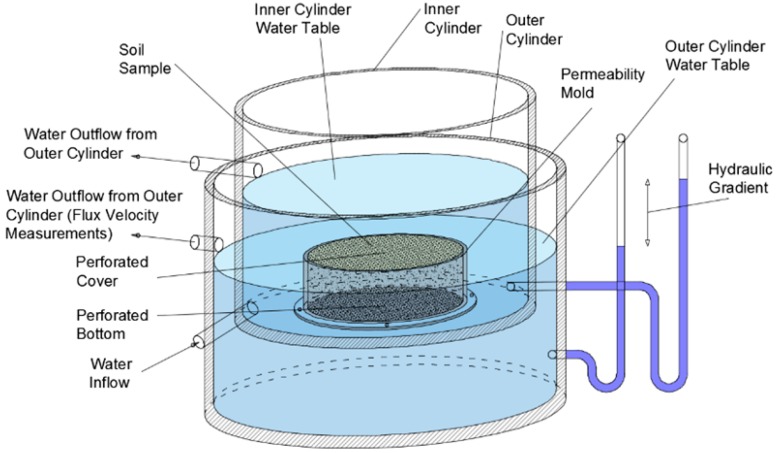
The permeameter scheme.

The permeameter construction (Figure 4) consists of internal and external cylinders made of stainless steel. Dimensions of the inner cylinder were: height h = 0.17 m, diameter d = 0.205 m, and the dimensions of the outer cylinder were h = 0.27 m and d = 0.19 m. Cylinders were connected by a permeability cylinder with a perforated bottom, also made of stainless steel, where the sample was placed. Then, after installing the sample, a perforated cover was placed on top. The permeability cylinder was attached to the inner cylinder by means of four screws and a rubber ring to ensure that there was no unexpected water leakage from the external cylinder. The principle of operation of this device is based on communication vessels that allow the flow of water from the external cylinder to the inner cylinder through the soil sample. The hydraulic gradient was simply determined by the difference between the outer and inner table heights. In practice, the internal water table is stationary and the hydraulic gradient is caused by the variable height of the external water table. The tests were carried out when both the internal and external water table were in a fixed position. Measurements of outflow water were repeated five times for each test point [31,45,46].

### 3.3. Estimation Theory

The theory of estimation concerns the inference of the correct probability distribution of the general population on the basis of independent variables from the tested sample. Using the knowledge about the distribution of classes in the test sample, an inference is made to the general sample. Parametric estimation occurs when the elements of the class of possible distributions of the general population differ only in the values of parameters. Non-parametric estimation is used in more complex cases when the differences in elements of the class of possible distributions of the general population concern not only the values of parameters, but also the form of the distribution function [47,48].

There are two parts of the theory of estimation: point estimation and interval estimation. The point estimation finds the function sampling and its value is taken as the best estimate of the value of the parameter for the overall sample. The interval estimation on the basis of the sample determines a numerical interval, which contains the value of a parameter of the general population, taking into account the assumed probability [48].

An estimator, with the general form given in Equation (1), is a statistic that serves to estimate the value of the distribution parameter with a function in the sample. It can be used to estimate an unknown parameter in a population.

(1) $$\hat{Q_{k}}=f\left(X_{1},X_{2},X_{3}\ldots ,X_{n}\right)$$

In the theory of estimation, there are three main features, which should be met by any good estimator to be useful for the created model. It should be unbiased, consistent, and effective [47,49]. The unbiased estimator is characterized by the realization of a random variable *X* comprising an N-elemental sample, which meets the condition:

(2) $$E(\hat{Q_{k})}=q_{k}$$

where: $\hat{Q_{k}}$ is an estimator, and $q_{k}$ is a parameter for a random variable *X*.

Estimators meeting this relationship are called unloaded estimators and the load of the estimator $\Delta \hat{Q_{k}}=E(\hat{Q_{k})}-q_{k}$ is equal to zero.

The estimator is consistent if an unlimited increase in the sample size occurs. The estimation $\hat{Q_{k}}$ of the parameter *q_k_* strives for a true value with a probability of one.

(3) $$P\left(\hat{Q_{k}}\to q_{k}\right)=1\;\mathrm{for}\;N\to \infty$$

The effective estimator shall be that which shows a smaller dispersion of the values obtained from all possible samples. The measure of spread is, therefore, the variance, and the smaller the variance, the better the unloaded estimator [49,50].

## 4. Test Results

The aim was to create a model that allows for determining the permeability coefficient, k, based on the physical parameters of the tested material. Analytical methods using statistical tools are widely used in scientific research, saving time and money. In this case, Statistica® (version 13, TIBCO Software Palo Alto, CA 94304 USA) was used as a statistical analysis tool. Preparation of the model was preceded by the collection of an appropriate database. The research hypotheses were formulated and preceded by an in-depth analysis of the phenomenon with the reference of parameters to each other. It is good practice to divide the sample into control groups and research groups, which allows for independent verification of the model. 

In the case of the studied phenomenon of permeability, the soil and test properties taken into account in the further analysis were grain size, gradient, porosity, specific density, and optimum moisture content. The values of gradients taken in this study were 0.2, 0.3, 0.4, 0.5, 0.58, 0.67, 0.75, and 0.83. The distribution of uniformity for the hydraulic gradients and granulometric compositions of the tested samples should also be emphasized. There were 20 independent tests for each granulometric compositions and for each gradient. The total sample was N = 640.

Initially, a preliminary statistical analysis of the conducted tests was carried out, in which the following descriptive statistics were evaluated for the parameters: mean, standard deviation, and standard error (Table 1).

Then a correlation analysis of the coefficient of permeability in terms of all physical parameters was carried out. Correlation results are presented in Table 2.

The parameters with the highest degree of correlation are marked in Table 2 in yellow. Correlations of material physical properties with the coefficient of permeability were the highest for granulometric coefficients, *d*_5_ and *d*_90_, specific density *ρ_d_*, and optimal water content *w*. All analyzed correlation coefficients were statistically significant with *p* < 0.05.

**Figure 5:**
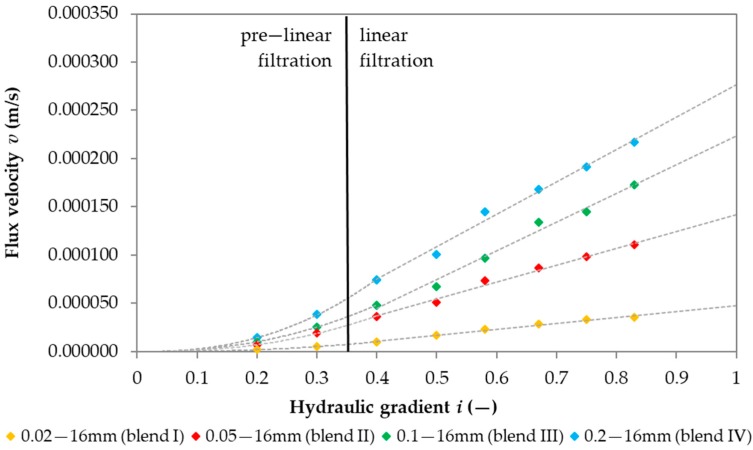
Results of average velocities of flow to a given gradient for different grain sizes.

The next stage was to examine the distribution of the flow velocity in relation to the hydraulic gradient for different blends included in the study. This was a dependence characteristic of the studied phenomenon. After examining the characteristics of the flow velocity distribution in relation to the gradient, it was found that the dynamics of the flow velocity changed with the gradient. For gradients above 0.3, it was linear. With gradients from 0.2 to 0.3, dynamics were slowed down and the changes had a pre-linear character (Figure 5), which means pre-linear filtration. Such a procedure allows for formulating a hypothesis on the possible need to create not only one common model to determine the permeability coefficient at any gradient but to separate equations into two phases of flow velocity. This had a significant impact on the determination of models for the permeability coefficient, and in particular, on the determination of independent variables.

The best convergence effect of the expected value in relation to the tested one was obtained using the non-linear estimation method. A series of tests were carried out based on previously selected parameters (variables) that were best correlated with the permeability coefficient. In the case of the attribute pairs with a high correlation coefficient (above R^2^ = 0.6), having a similar influence on the permeability coefficient, one of the attributes was omitted. This was to improve the reliability of the regression model and to ensure the stability of the parameter estimation of this model.

The highest variance coefficient with R^2^ = 0.615, was obtained using the following:

(4) $$k=\left(\rho_{d}\times \left(-0.00122\right)\right)-(\left(-0.0022955\right)\times {\left(d_{5}\times d_{90}\right)}^{0.09942}$$

The assessment of fixed parameters for the model is included in Table 3.

**Figure 6:**
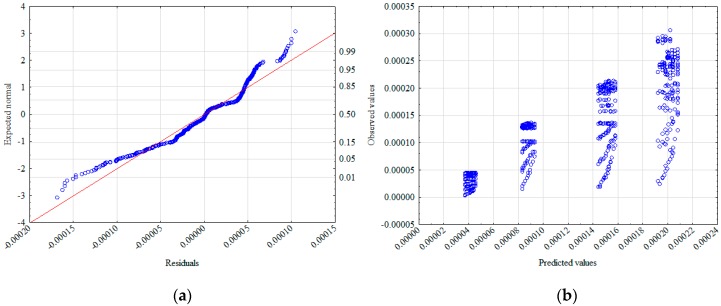
Normality diagram of residuals (**a**) and values observed against predicted (**b**).

To verify the hypothesis of the effect significance for the two phases of flux velocity in relation to the hydraulic gradient, further statistical analysis was performed. An analysis of the normality of the residue distribution (Figure 6a) and the distribution of the observed values in relation to the predicted ones (Figure 6b) were prepared. Both analyses supported the belief that it is reasonable to adjust the model to include both pre-linear and linear phases. In order to maintain the universality of the designated model, it was decided to adjust only the independent variables to it, while retaining the dependent variables used in model 1 (*ρ_d_*, *d*_5_, *d*_90_).

For the pre—linear phase (gradients of 0.2–0.3), after the determination of independent variables, the explained variance was obtained at the level of R^2^ = 0.622. The values of the independent variables, together with the description of their standard errors, are described in Table 4.

**Figure 7:**
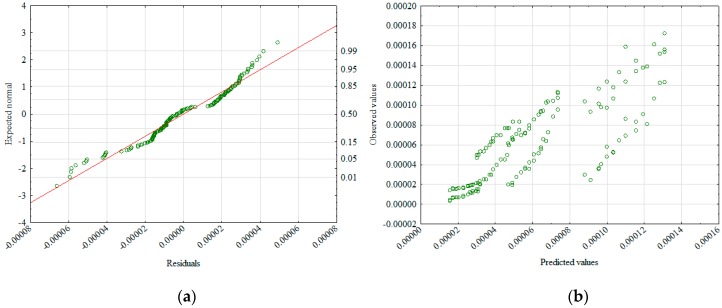
Normality diagram of residuals (**a**) and values observed against predictions (**b**).

The new formula (Equation (5)) is characterized by a better matching of observed values in relation to the calculated values (Figure 7a,b), but also by a better distribution of residual values.

(5) $$k=0.00173+\left(\rho_{d}{}^{0.0259}\right)-\left({\left(d_{5}\times d_{90}\right)}^{0.00004}\right)$$

A further step was to determine the independent variables for gradients 0.4–0.83 for the linear phase, where the explained variance was R^2^ = 0.883. Table 5 shows the determined independent variables, which also contains parameter values together with standard errors for the determined variables.

**Figure 8:**
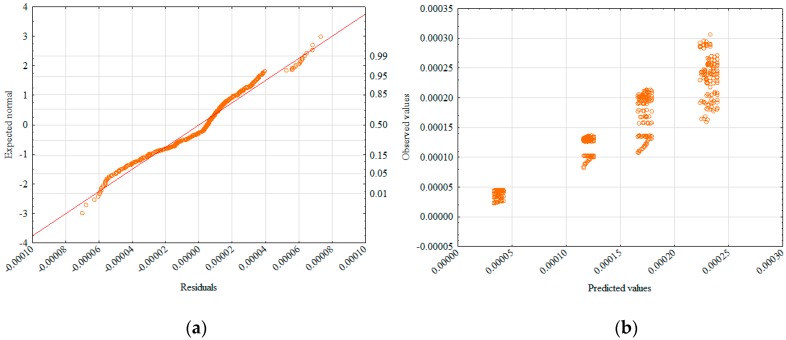
Normality diagram of residuals (**a**) and values observed against predicted (**b**).

The correctness of the model (Equation (6)) was supported by the diagram of the normality of residuals distribution (Figure 8a) and the distribution of observed values in relation to predicted values (Figure 8b).

(6) $$k=\left(-0.001169\times \rho_{d}\right)+\left(\frac{0.00165\times d_{5}}{d_{90}{}^{-0.163}}\right)+0.00185$$

## 5. Result Discussion and Conclusions

**Figure 9:**
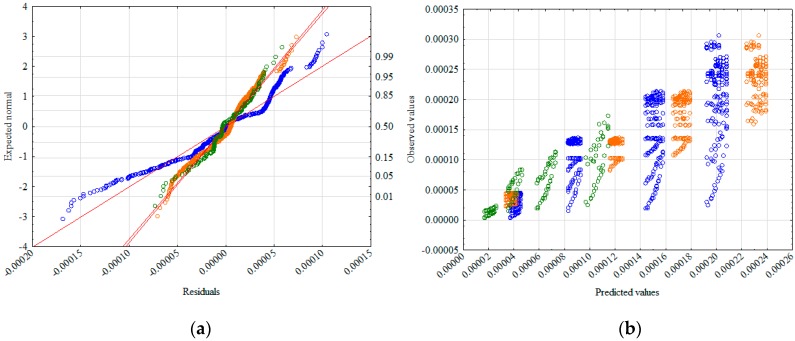
Normality diagram of residuals (model 1—blue color, model 2—red color, and model 3—green color) (**a**) and values observed against predictions (model 1—blue color, model 2—green color, and model 3—orange color) (**b**).

Determination of new independent variables and the application of pre-linear and linear phases allowed for estimating the calculated flow velocity results. This was not ensured by the first solution but fulfilled the assumptions of the hypothesis presented in the article. At the same time, the importance of accounting for the division into phases when determining the flow velocity was confirmed. This is best illustrated in Figure 9a, which shows the residual normality distributions for all models. By dividing the phases, a significantly better result of the explained variance for the linear phase was achieved. A better matched model for the pre-linear phase was also found, resulting in an increased accuracy of the resulting model. This is illustrated in Figure 9b, which compares the values observed and calculated for all models.

The parameters considered when creating the models were used to create the limits of applicability of the model by means of basic descriptive statistics. For each parameter (variable) used, the mean, standard deviation, and minimum and maximum value were calculated. The results are presented in Table 6.

The presented data defines the limits of applicability of the designated models. The conclusions are summarized below:

- The RCA material tested for blends with 0.02–16 mm, 0.05–16 mm, 0.1–16 mm, and 0.2–16 mm grains was characterized by good permeability from 3.1 × 10^−4^ to 4 × 10^−6^ m/s. Reported coefficients of permeability by Azram and Cameron [51] of RCAs with gradation in the range of 0–20 mm with 6 to 7% fine particles (*d* < 0.0063 mm) were in the range of 2 × 10^−7^ to 2 × 10^−8^ m/s. Arulrajach et al. [52], based on constant head test experiments, estimated the coefficient of permeability in range of 2.04 × 10^−3^ to 3.3 × 10^−8^ m/s, which indicates the existence of non-Darcian flow. Tests performed by McCulloch et al. [53] were conducted for RCAs with 4% fines and with fractions of 0–50 mm. The reported coefficients of permeability were in the range of 1 × 10^−4^ to 3 × 10^−4^ m/s. Tests on RCAs with poorly graded RCAs with fractions of 6–12 mm and with no fine content have proven a high water permeability of such material with a coefficient of permeability of approximately 1 × 10^−3^ m/s [53]. Tests performed on an RCA blend with gradation of 0−50 mm and with 5% fine contents have shown that the coefficient of permeability calculated based on a constant head permeability test is equal to 1.06 × 10^−6^ m/s [54]. As can be seen, the coefficient of permeability value strongly depends on the fines content. Test results presented in this article corresponds with the test results presented by other studies.
- The specific density, optimal moisture content, and particle sizes *d*_5_ and *d*_90_ had a significant influence on the determination of the permeability coefficient.
- Regarding RCAs, a relationship between the flow velocity and the hydraulic gradient showed the existence of two phases, namely pre-linear and linear.
- Models created for individual phases gave greater confidence in determining the permeability coefficient.
- The models were created on the basis of the same set of variables, which facilitated their application and implementation in practice.
- Each of the models was examined in terms of the discrepancy with the observed value in relation to the forecasted results.
- For each of the models, the limits of its applicability were estimated.

The solutions presented here indicate the possibility that their use in calculating the flow rate was particularly advantageous due to the lack of such characteristics for this type of materials. To properly design a geotechnical structure, it is important to have information about geotechnical parameters and the solution presented here provides the basis to receive them.

## Figures and Tables

**Table 1:** Average test against a fixed reference value.

Variable	Description	Mean	Standard Deviation	Standard Error	Test t
*k*	Coefficient of permeability	0.000120	0.000078	0.000003	38.958
*v*	Flux velocity	0.000072	0.000061	0.000002	29.890
*ρ*	Specific density	1.931437	0.019769	0.000781	2471.586
*n*	Porosity	0.374610	0.083305	0.003293	113.762
*e*	Void ratio	0.611371	0.190529	0.007531	81.177
*w*	Moisture content	0.105425	0.009157	0.000362	291.260
*d*_5_	Particle size when passing 5%	0.230000	0.018723	0.000740	310.774
*d*_10_	Particle size when passing 10%	0.287500	0.013001	0.000514	559.457
*d*_30_	Particle size when passing 30%	0.837500	0.086200	0.003407	245.793
*d*_60_	Particle size when passing 60%	4.675000	0.334739	0.013232	353.318
*d*_90_	Particle size when passing 90%	9.350000	0.753915	0.029801	313.747

**Table 2:** Correlation table for coefficient of permeability k (m/s).

Variable	Value of Correlation for Coefficient of Permeability *k* (m/s)
*i* *	0.504794
*ρ*	−0.754960
*n*	−0.112145
*e*	−0.137592
*w*	−0.737912
*d*_5_	0.772737
*d*_10_	0.569209
*d*_30_	−0.140365
*d*_60_	0.408963
*d*_90_	0.701564

* *i*—Hydraulic gradient.

**Table 3:** Evaluation of fixed parameters for model 1.

Independent Variable’s Label	Independent Variable’s Value	Standard Error
a1	−0.001220	0.000231
b1	−0.002296	0.000464
c1	0.099420	0.029056

**Table 4:** Evaluation of fixed parameters for model 2.

Independent Variable’s Label	Independent Variable’s Value	Standard Error
a2	0.001734	0.000319
b2	−0.002594	0.000455
c2	−0.000040	0.000030

**Table 5:** Evaluation of fixed parameters for model 3.

Independent Variable’s Label	Independent Variable’s Value	Standard Error
a2	−0.001169	0.000150
b2	0.00165	0.000256
c2	−0.163	0.058118
d2	0.00185	0.000307

**Table 6:** Parameters of applicability of the formula.

Variable	Mean (m/s)	Std. Dev.	Minimum (m/s)	Maximum (m/s)
*k*	0.000120	0.000078	0.000004	0.00031
*ρ_d_*	1.931438	0.019769	1.895000	1.95600
**d_5_	0.230000	0.018723	0.200000	0.25000
*d*_90_	9.350000	0.753915	8.600000	10.20000
